# The (Ir)Responsibility of (Under)Estimating Missing Data

**DOI:** 10.3389/fpsyg.2018.00556

**Published:** 2018-04-20

**Authors:** María P. Fernández-García, Guillermo Vallejo-Seco, Pablo Livácic-Rojas, Ellian Tuero-Herrero

**Affiliations:** ^1^Facultad de Psicología, Universidad de Oviedo, Oviedo, Spain; ^2^Faculty of Humanities and the Faculty of Medical Sciences, Universidad de Santiago de Chile, Santiago, Chile

**Keywords:** missing data, warnings, recommendations of the experts, advice of the experts, sensitivity analysis, prevention

## Abstract

It is practically impossible to avoid losing data in the course of an investigation, and it has been proven that the consequences can reach such magnitude that they could even invalidate the results of the study. This paper describes some of the most likely causes of missing data in research in the field of clinical psychology and the consequences they may have on statistical and substantive inferences. When it is necessary to recover the missing information, analyzing the data can become extremely complex. We summarize the experts' recommendations regarding the most powerful procedures for performing this task, the advantages each one has over the others, the elements that can or should influence our choice, and the procedures that are not a recommended option except in very exceptional cases. We conclude by offering four pieces of advice, on which all the experts agree and to which we must attend at all times in order to proceed with the greatest possible success. Finally, we show the pernicious effects produced by missing data on the statistical result and on the substantive or clinical conclusions. For this purpose we have planned to lose data in different percentage rates under two mechanisms of loss of data, MCAR and MAR in the complete data set of two very different real researchs, and we proceed to analyze the set of the available data, listwise deletion. One study is carried out using a quasi-experimental non-equivalent control group design, and another study using a experimental design completely randomized

The evaluation of the efficacy of the administration of a clinical treatment, or of a component of the clinical treatment, whether to resolve a physical or psychological health problem or a behavioral dysfunction, often involves the registration of different variables that indicate the treatment effect at all of the moments necessarily involved in its administration. It also involves their registration in different phases of follow-up in order to examine whether the results achieved are maintained over time or not. However, it is not always possible to obtain all of the measures (e.g., López et al., 2016; Turan et al., 2016; Cano-García et al., 2017; Gathright et al., 2017). Data on the variables that it has not been possible to record despite having planned to obtain their value (Carpenter and Kenward, 2013) are called “missing data,” and are the objective of this study.

Missing data can occur at any time when carrying out any empirical research, and to a large extent, the more subjects we have, the less control we can exercise over them, the longer duration of the investigation, the more variables we record at each moment in time throughout the entire duration of the research, and the more distanced the records are (the aforementioned investigations attest to this). Missing data are *always* a problem, the severity of which will depend on the cause and the amount of data that is not available, and they could even invalidate the research. Therefore, it is advisable to be proactive and to understand what the possible causes are, how they manifest in our data matrix, what the consequences are, what the alternative solutions are, and what else we can do to minimize, as much as possible, the likelihood of this problem occurring in our research and any harm it could cause.

## How missing data manifest and their probable causes

It is possible that for some subjects we only know their identification, or we lack the record of some variables simply because they did not occur naturally, or because the measuring instrument was insensitive to capture them, or due to the poor formulation of variables or a lack of data because the researcher removed them for some reason, or has neglected the record, or the record is wrong, or the sampling was unsatisfactory. All of these situations are specific cases, with specific solutions that we are not going to cover here. Thus, we feel it should be made clear that we start from the assumption that the person solely responsible for the answer not being recorded is the subject, and they may not have provided it voluntarily or involuntarily.

When the missing data do not have any relation to the actual or potential study variables (e.g., when they are due to a transfer of residence, to the forgetting of an appointment, or any other unforeseeable cause outside of the study), the losses are considered to be *completely random*. If data is not recorded due to issues related to the biological, psychological, social and/or cultural diversity of subjects, or for reasons arising from the effect of the treatment administered impeding the effective recording of the variable of interest, or impeding attending the appointment due to the deterioration of the necessary functions to issue the response, it is considered that the non-issuance of the response is due to *random causes*. In these cases the non-issuance of the response appears randomly among the subjects suffering from these particular circumstances, however, these causes are not completely random because they can, to some extent, be predicted by the researcher (e.g., it is expected that subjects who worsen after receiving treatment are more likely to leave the study than those who improve). The researcher is able to plan the recording of the variables that can explain the loss of these data. Sometimes subjects do not provide certain answers with the intention of preserving their privacy, either due to embarrassment, or because they simply wish to hide the truth. The reasons may be diverse, for example because they wish to disguise or conceal their wealth, because they have failed to adhere to the treatment protocol at a specific moment, because they are not motivated to receive the treatment, etc. All of these causes involve some underlying process that is somehow related to the timely measure of the variable that we are recording or the process of change over time that we are analyzing. As we neither have nor know any variables that explain the absences, we can only assume that they depend on the variable recorded itself. These unknown causes that determine the *non-response* of subjects are always non-random and are the most dangerous for inference.

The interest in missing data is old (Wilks, 1932), but the relevance of the problem was not recognized in all its magnitude until Rubin (1976) formalized, in statistical and mathematical language, the causes outlined above, elevating them to the role of *mechanisms* or processes responsible for the loss of data called MCAR, MAR and MNAR, *missing completely at random, missing at random* and *missing not at random* respectively, as we have described in the preceding paragraph. When the loss is caused by a MCAR or MAR mechanism, the probability of missing observation, given the observed data, is the same for all observations of the sample in the first case, or a specific subsample in the second. In these cases, statisticians refer to it as a non-informative or ignorable loss. However, when the loss is due to a MNAR process, the conditional probability of the missing data, given the observed data, is not the same for all of them, and it is directly dependent on the variable that is being recorded. The experts define this loss as informative and non-ignorable. After the abovementioned examples, it is easy to understand why. In any of them the loss pattern displays as intermittent or monotonous.

## The consequences of data loss

The only way of knowing the consequences of data loss and their severity is through controlled experiments manipulating the data loss in the form of the mechanisms described by Rubin. This has been the means, by computer simulation, or using entire databases from real investigations, that has allowed us to verify that data loss is rarely innocent, and that the cost is both statistical and substantive. To sum up, the following are the five main consequences:

First: the representativeness of the population in the sample will disappear and the transferability of the results will be limited.

Second: selection bias. The data we lose contain important information and will inevitably lead to selection bias (Meng, 2012). The size of this bias will depend on the amount of data missing, obviously, but especially on the particular association of the incomplete records with the records of other variables, including the particular dependence the incomplete records have with the unknown values of the same variable in which they occur.

Third: statistical analysis techniques lose their effectiveness (multivariate techniques that require complete data would not even be implemented). Namely, the normal distribution of the data may not be maintained. Nor the homogeneity. The variability of the data will increase or decrease, and with it the standard error will increase or decrease seriously damaging the estimation of the parameters that in some occasions will be overestimated and in others will be underestimated.

Fourth: loss of data means loss of sample, and consequently involves the loss of power of the test.

Fifth: although not all data that are missing carry the same load and quality of information, analyzing data that have poor or impoverished information will make the researcher choose an estimation model that does not respond to reality because it omits relevant variables or includes irrelevant variables.

These five powerful reasons have concerned scientific organizations and drug regulatory agencies worldwide. In fact, since the 1990s, these entities have not ceased to give warnings or to offer recommendations for how to manage the problem of missing data in research. For example, the U.S. Food and Drug Administration (FDA) had called on the National Research Council (NRC) to bring together the most outstanding statisticians and mathematicians on this subject with the mission to provide guidelines for researchers, both to limit the data loss and to address the problem if it has already occurred (National Research Council, 2010). Today, there is no branch of science that is not concerned with this issue, and there are researchers who treat this issue with the importance it deserves (e.g., Fielding et al., 2016).

## Ways to proceed when our data matrix is incomplete due to any of the reasons described above

We believe it is important to highlight, firstly, that it is not always appropriate to intervene to address data loss. If the loss is small or too large, one should not intervene in the first case as it is unnecessary, and in the second case because it would be reckless. It has been shown that if the data loss is no more than 5%, any technique that we use, whether simple or sophisticated, will lead to the same conclusions as those that would be found if we did not include the subjects whose data vector is incomplete (Little and Rubin, 2002). It goes without saying that the latter option is the most reasonable solution in these cases. There have been no explicit manifestations with regards to the maximum amount of data that can be missing while the available techniques are still effective in making use of the information that remains in our database and leading to valid conclusions (Meng, 2012). Thus it would be most sensible and appropriate to make a critical evaluation of the situation. If the data loss is very high (e.g., Collins et al., 2001 consider it high if it is greater than 40%) it would be reasonable to conduct the investigation again. Look for reasons for this, and surely you will be thinking the same as us: there has been negligence and carelessness on the part of the investigator, the measuring system for the variables has not been chosen adequately, the times of recording have not been respected, etc., all reasons with enough weight to invalidate any investigation.

No one will dare to state when a rate of data loss is sufficiently small that the consequences will not be felt (Schafer, 1997), but the controlled experimentation mentioned above has shown that problems resulting from missing data become serious when the figure is around 10% (Little et al., 2014; Li et al., 2017). Thus if, despite our efforts in taking care of the design and data collection, we have lost more than 5% of the data, but not such a large amount as to ruin the investigation, we must proceed *to intervene to retrieve the information*. As this is not an easy task, it is prudent to take into account the warnings, recommendations and advice of the experts, especially if the consensus is virtually absolute.

### Warnings

There are two practices that are most commonly used to treat the missing data: eliminating subjects with incomplete data (listwise deletion), and imputing (assigning a value to) the missing data with the mean of all of the values or with the prognosis derived from the regression analysis. However, these are almost never appropriate ways of proceeding (Rubin, 1987; Schafer, 1997; Schafer and Graham, 2002; Enders, 2010; Graham, 2012; Meng, 2012). As noted by Ato and Vallejo (2015), these two methods were the only way to deal with non-informative losses in the majority of professional statistical programs until relatively recently, but it has been proven that when proceeding by listwise deletion we lose sample, and with it information and power of the test, and when it is done by imputing the marginal mean (of the variable) or the conditional mean (prognosis by regression) a number of analytical problems occur (remember the outstanding consequences on the *third* of the undesirable effects) just by adding data (constant value) without adding information (Enders, 2010). The representativeness of the population is likely to be compromised when one proceeds in either way. None of these techniques take into account the relationship the incomplete variable has with all of the other variables, or they take it into account in a very limited way (simple imputation by regression), and therefore they would *only* be a possible option (as a solution and therefore, of choice) if the loss were *completely at random* (MCAR). However, when the loss reaches 10% or greater, the possibility that it is *completely at random* is very unlikely, and if it were, the aforementioned consequences of using the two methods would be unavoidable. However, the loss may be *at random* (MAR) and therefore ignorable as well. Today, experts agree that the most recommended procedures for treating ignorable missing data, which are also implemented in all professional statistical packages, are multiple imputation, and maximum likelihood estimation (National Research Council, 2010). We will now go on to discuss these techniques. It should be noted that sometimes the methods of inference based on quasi-likelihood (e.g., the approach of generalized estimating equations) are also a reasonable option (see Vallejo et al., 2011).

### Recommendations

It is absolutely necessary to examine how missing data are distributed in the database to try to figure out which mechanism was responsible for the loss and how it was produced. The purpose is to determine whether the data loss is ignorable (MAR and MCAR) or non-ignorable (MNAR), and what the pattern that is made looks like, whether monotonous or arbitrary. Based on these two aspects we must choose the most appropriate technique for dealing with the loss of information. Other aspects, such as the number of variables in which there is loss and what type of variables they may be, are always subordinate to the above two considerations. The most appropriate option will be any of the following that we shall now discuss:

A. When we can determine that the data loss is MAR we are assuming that it is possible to recover the missing information taking advantage of all of the information contained in the cases that are complete. We can do this in two main ways: through methods based on direct estimates of maximum likelihood and using techniques of multiple imputation. Let's take a look.

A.1. — Methods that are developed directly maximizing the likelihood function (ML). These methods do not fill the gaps in our database, but they calculate the parameters that make the data more credible, maximizing the likelihood function of the complete data.

Many studies testify that longitudinal studies are the most common when the purpose is causal, and that repeated measures designs are most commonly used to collect the data (Fernández et al., 2007; Arnau et al., 2010). Although these designs can cover various objectives, often the interest is in testing the efficacy of a treatment and assessing the evolution of the behavior experienced over a period of time. Sometimes it is intended only to make population inferences, but sometimes we are interested in examining the individual behavior from the responses of each subject (growth curve analysis, see Blozis and Harring, 2015; Blozis, 2016). The most sensible thing to respond to both types of hypothesis is to analyze the data using the mixed linear model (MLM), or, if we also have latent variables, using Structural Equation Modeling (SEM). Both MLM and SEM absorb the General Linear Model and are far superior to it allowing us to study nested models, introduce variables that change over time, consider different correlation structures, etc. They even allow us to carry out parameter estimates when we do not have a complete record of all of the subjects (see Fernández et al., 2007) and the variance of the random effects of the nested model are heterogeneous (Vallejo et al., 2015). These latter aspects are the ones that interest us in this study.

MLM and SEM models perform the maximum likelihood estimates of the parameters using iterative algorithms. The most efficient, and the most popular too, are the EM (Expectation-Maximization) and FIML (Full Information Maximum Likelihood) algorithms in the MLM and SEM models respectively. They take into account all of the information contained in the complete data, so if the data loss is MAR, incorporating the cause of the loss into the analysis model provides unbiased estimates of the parameters (Little and Rubin, 2002; Enders, 2011).

A.2. — Multiple imputation methods. In the variables that are recorded on several occasions there is, obviously, a greater chance of losing data and it is always possible to assemble the missing data from a monotonous pattern of loss. In addition to the main variable of the study, in repeated measures designs it is common to record many other variables that may moderate, mediate or confuse the relationship studied, for example, quality of life, motivation, self-regulation, resilience, etc. (MacKinnon and Luecken, 2008; Loeys et al., 2015). These variables are often recorded less frequently than the variables that are indicative of the treatment effect. In fact, it is customary to record them only before and after the intervention. At other times, the research is genuinely cross-sectional, with the aim of studying the possible direct and indirect relationships between the variables and they are only recorded on one single occasion. Immersed in a longitudinal or transversal research, these data may be missing, and absences can be organized according to a monotonous or arbitrary pattern of loss. For these other situations we can choose a multiple imputation procedure.

Multiple imputation (MI) deals with missing data in three steps. First several imputations (plausible values) are performed for each missing datum in order to have as many replications of the data sample as the number of imputations we carry out. Then each data set is analyzed in the necessary way in order to answer the research hypotheses. Finally all of the results are combined into one using the formulas developed by Rubin (1987) or Schafer (1997).

Of the three steps, the most delicate one is the first, the generation of the imputations. Two things matter the most here: the quantity and quality of the imputations. As for the amount concerned, not so long ago it was considered that between three and five, or no more than 10 imputations (Schafer, 1999) were sufficient for the estimates to be valid. Today, however, most authors argue that the more imputations the better, at least as many as the cardinal number that describes the percentage of missing observations (White et al., 2011).

The imputed values are of quality if they are consistent with the values of the variable on which they are performed (the original distribution is not altered) and also with the other variables (the correlations between them are not altered). This can only be achieved if the imputation model is able to capture the true structure of the data, and for this it must necessarily contain the following. Firstly, in addition to the variables of theoretical interest, it must include auxiliary variables, that is, variables associated with the loss mechanism, and all those that are correlated with the previous variables (Carpenter and Kenward, 2013; Enders, 2016; Kontopantelis et al., 2017). The first are absolutely necessary, the second make the MAR assumption plausible, and the third help estimate the scores more accurately. Secondly, it is important that the imputation model is at least as complex as the analysis model. For example, suppose a researcher is interested in testing an interaction between two variables (X1 and X2) in a regression analysis with missing data. In this case it is important to incorporate into the imputation model the effect X1 and X2, but also the product of the two predictors (Enders et al., 2014).

There are several ways to perform the MI, depending on whether the loss pattern is monotonous or arbitrary. When it is monotonous it is done using regression techniques. When the loss pattern is arbitrary, the MI is performed by the Markov Chain Monte Carlo (MCMC) procedure. This procedure contains two great properties. Firstly, it uses the power of Bayesian inference to estimate the posterior probabilities of the responses. This procedure is powerful for two reasons: it works on the likelihood function, albeit indirectly, by imposing a prior distribution to the variables and, in addition to the information contained in the sample, it can incorporate external information. Secondly, once the most plausible distribution has been obtained for the data that are complete, by Monte Carlo random sampling procedures, all of the desired imputations are made for each missing datum.

The high power of the techniques outlined in paragraphs A.1 and A.2 lies in two aspects that they have in common. First, they assume that the probability of complete data can be estimated from the observed data by controlling the effect of the missing data. This assumption is valid when the loss mechanism is MAR (Rubin, 1976), and these techniques proceed accordingly, taking into account all of the available information in the database. Second, they are developed based on iterative processes (maximum likelihood, directly or indirectly). As many iterations are carried out as are necessary until *convergence* is reached, i.e., until the maximum possible accuracy is reached in the parameter estimation (ML), or approximating the posterior distribution of the data (MCMC), or approximating the best prediction model for estimating the data (regression techniques).

However, these are different techniques with different characteristics and they obviously have their own properties which represent advantages over one another in certain circumstances. At some point, the researcher needs to decide which technique is more prudent to choose, regardless of the custom or expertise he or she may have. The following techniques are noteworthy:

Techniques of multiple imputation: these allow the imputation and analysis models to be different, thus, once the imputations have been done it is possible to carry out as many statistical analyses as you consider appropriate to test the relevant hypotheses without making imputations repeatedly in each analysis. Added to this, if we perform the imputations using techniques with Bayesian support we add even more value for three reasons: it is easy to introduce as many auxiliary variables as we consider appropriate; it is possible to incorporate external information (both are notable for gaining efficiency and accuracy values); and, due to the mechanism that approximates the posterior distribution, they are techniques that are robust to the violation of the assumption of normality.

Techniques that work directly on the likelihood function: these allow the comparison, using the likelihood ratio and/or information criteria of as many models as we consider reasonable. The advantage of adjusting random effects models, mixed models, and hierarchical linear models, whether the designs are repeated measures or not, balanced or unbalanced, with loss of data or complete data is noteworthy (Fernández et al., 2007). Also worth noting is that the power of the test is similar to that which would have been obtained with the full data set because it does not take into account the uncertainty associated with the likelihood function; it only considers the uncertainty associated with the estimation of parameters.

Despite the differences, it has been demonstrated empirically that the two procedures are equivalent in resolution when the sample size is large, the data loss is moderate, and the distribution is normal (Vallejo et al., 2011). The experts agree that these two procedures are the most suitable methods to use when the loss mechanism is ignorable (MCAR and MAR; Little et al., 2014; Lang and Little, 2016). In some cases we can even use the two techniques together, thus capturing the benefits of both. For example, when it is appropriate to impute data using MCMC, we can impose a priori the distribution resulting from using the EM procedure. Some authors argue that this is the best option (Enders, 2010).

B. — If, after detailed examination of the data matrix we conclude that the mechanism responsible for the loss of our data *is not completely at random*, and we rightly assume that a MAR or MNAR mechanism is more reasonable, our data analysis is complicated because formally there is no way to discern between one or the other, and if they really were MNAR, the abovementioned alternatives provide biased estimates.

To try to overcome this problem, it is recommended to use, together with the abovementioned procedures that assume MAR models, one or more of the MNAR models available, such as *Selection Models, Shared Parameter Models and Pattern-Mixture Models* (an excellent explanation of these procedures can be found in Enders, 2011; Gottfredson et al., 2014), and to evaluate the impact of different loss patterns by performing a *sensitivity analysis* (Blozis et al., 2013).

Such an analysis should always be based on adequately supported (clinically plausible) substantive hypotheses which faithfully reflect the pattern produced by the loss mechanism that supports these hypotheses. Once we have obtained the results of all of the models we have tested, we must compare them primarily based on the bias, accuracy and coverage, and conclude with the best. For example, there is currently a broad consensus that it is most appropriate that the primary analysis of longitudinal data in clinical settings is effected with methods which assume MAR missing data, and that the robustness of the results obtained in this way is evaluated by sensitivity analysis using methods which assume MNAR missing data (National Research Council, 2010). Therefore, the main objective of the sensitivity analysis is to determine whether the conclusions (inferences) referring to the treatment effect under MAR methods are reversed when MNAR methods are used. The smaller the differences between the compared results, the more our confidence increases in assuming that the missing data are MAR.

Because of the complexity of research projects today, researchers should listen to the advice of experts, which we summarize below:

### Advice

If there is missing data it greatly complicates the task of analyzing the data for two main reasons. One reason is that the most robust techniques mentioned above are complex and carrying them out correctly involves great difficulty. The other reason is that there is no universally valid way for all situations, and succeeding in choosing the most appropriate procedure to retrieve information from our data often requires the skill of an expert. Both are compelling reasons to help us understand the insistence of mathematicians and statisticians (experts in the development of techniques to deal with this major problem of missing data) on the following four aspects:

First: the best solution is not technical or analytical, but tactical. We are referring to prevention. It is mandatory to take care in every aspect of the research and throughout the entire process, aiming, through the use of design strategies and with great persistence, to minimize every possible chance of losing data (National Research Council, 2010).

Second: if we take care with the design, we extend the window of opportunity to ensure the soundness of the inferences, because we can see which variables determine the subjects' responses and which variables determine their absence of response and we can include them as auxiliary variables in the models of imputation and/or analysis, making a MAR model more plausible. This renders the treatment of the data more successful (Little et al., 2014; Lang and Little, 2016).

Third: the process we use to solve the problem is not always a guarantee of a successful outcome. The solution will always be uncertain. We must not make the mistake of thinking that we have adequately addressed the problem, not even using the most sophisticated procedure possible. To date, there is no foolproof way that allows us to discern with absolute certainty whether the loss mechanism is MAR or MNAR, nor is there a method that allows us to reproduce the original data, nor can we be sure that the model we propose allows us to capture all the fine details that underlie the loss mechanism. For all these reasons it is highly recommended always to perform a sensitivity analysis (Enders, 2010; Graham, 2012; Carpenter and Kenward, 2013; Mallinckrodt and Lipkovich, 2017).

Fourth: although we have decided to eliminate subjects who have empty records because we believe it is the best justifiable choice, we must always recognize the problem, communicate it and discuss the reasoning of our decision (Lang and Little, 2016). The limited extension of a scientific article denies the opportunity to highlight the particularities of missing data and their treatment in more complex contexts (e.g., multilevel contexts), or when data is missing in categorical or ordinal variables rather than metrics. It is impossible to cover the entire spectrum of inferential methods and perspectives like Bayesian modeling, parametric likelihood inference, semi-parametric methods, non-parametric procedures including quasi-likelihood, empirical likelihood, and design-based weighting. To deepen one's knowledge of all of this, and to understand where the most research effort is being invested, we recommend consulting the missing data monographic issues of three journals, the *Journal of Statistical Software* (vol. 45, 2011), *Stat*í*stica Sinica* (vol. 16, 2006) and *Econometric Reviews* (vol. 33, 2014).

## Demonstration

In this section we show the pernicious effects produced by the loss of data on the statistical result and on the substantive or clinical conclusions. For this purpose we will act as if we lose data in the complete data set of two very different real researchs. Both researchs have two things in common, both have treatment group(s) and a control group, and in both cases the application of treatment has been extremely careful to guarantee the integrity of the treatment and internal validity, and data registration has been taken carefully with the purpose of not losing data. For this reason, both have the complete data set (with some nuances that we will detail later). We present both examples making use of the following sections: description of the research and objective, data analysis, results and conclusions with the complete data set, conditions of generation of lost data, and analysis, results and conclusions obtained with the set of the available data (listwise deletion) in the different data lost conditions. Finally, we present some molar conclusions and particular nuances about the empirical and substantive results derived from the loss conditions manipulated in both researchs. Both the analysis of the data and the loss of the data were made using the statistical package SPSS 25. Due to space limitations, results, tables referred to in the text, and added explanations of some paragraphs (indicated in the text as, Addenda 1^*^, 2^*^, etc.,) go in an attached file: Addenda. http://www.unioviedo.es/addenda

### First research

#### Description and objective

A non-equivalent control group quasi-experimental research was carried out with 3rd and 4th year primary school children to evaluate the effectiveness of an intervention to enable or reinforce self-regulation strategies in learning. This research was presented at the *5th International Congress of Educational Sciences and Development* held in May 2017 in two communications by a group of researchers from the areas of Education and Methodology of the University of Oviedo. In the first of them, Fernández et al. (2017) exposed the rigor with which it was carried out to avoid selection bias and guarantee internal validity (Fernández et al., 2014 show the difficulties and problems that may arise in quasi-experimental investigations and describe how they should be addressed). In the second one, (Livácic-Rojas et al., 2017) explained what tasks and strategies were used to avoid losing data.

Both experimental conditions, control group (CG) and treatment group (experimental group, EG), were randomized. 925 children from 14 schools in Oviedo participated. Before the application of the treatment, different tests were administered in collective sessions to know the initial state of the students in different competences, abilities and attitudes in which the treatment should have a positive effect. The treatment program involved 12 intervention sessions, one 60 min session per week. After the intervention, 915 students were evaluated again with the same initial instruments. The data of 10 students were lost in a completely random way due to the change of residence of their families.

#### Data analysis

The PROLEC-R Battery was one of the instruments used to evaluate the effectiveness of the intervention (Addenda 1^*^). Some of the results obtained with the analysis of the pre- and post-measures, hereafter PR and post PR, were presented in the mentioned communications, and are the only ones to which we are going to refer. The full results of the research are in the process of being published in different works.

The analysis of the pre-PR measurement showed no statistically significant differences between the experimental groups. The “gross” effect of the treatment on the post-PR measurement was tested by the Variance Analysis Model, ANOVA (2 × 2 × 2) [EG, CG; boys, girls; 3°P, 4°P]. The analysis of the change experienced between the post-PR and pre-PR measures and the analysis of the maturation effect was made by the ANOVA on the change scores (post-PR–pre-PR), hereafter ANOVA_ChS_ (2 × 2 × 2). When there are no initial differences between the EG and CG groups, the ANOVA on the Post measure and the ANOVA_ChS_ on the change scores are two types of analysis useful and valid in the non-equivalent control group quasi-experimental design (see Fernández et al., 2014). Both analyses have been carried out by adjusting models (see Ato and Vallejo, 2015) Addenda 2^*^.

#### Results obtained with the complete data

See Tables 2, 3 in Supplementary Material, first row. The ANOVA (2 × 2 × 2) showed that the additive model [T + S + C] best explains the results observed in the post-PR variable. Treatment [F_T_ = 145.98; *gl* = 911; η^2^ = 0.138; *p* = 0.000; 1-β = 1; MD ^*^EG-CC = 1.90] (empirical statistic F, error degrees of freedom, effect size, p value, power of the test and estimated mean difference, respectively). The asterisk is placed next to the initials of the group with the highest mean), Sex (Sexo) [F_S_ = 11.79; *gl* = 911; η^2^ = 0.013; *p* = 0.001; 1-β = 0.929; MD ^*^niñas-niños = 0.562], and academic Course [F_C_ = 25.81; *gl* = 911; η^2^ = 0.028; *p* = 0.000; 1-β = 0.999; MD ^*^4°P-3°P = 0.833].

The ANOVA_ChS_ (2 × 2 × 2) showed that the non-additive model Treatment × Course [T + C + (T × C)] explains best the change experienced between the post-PR and pre-PR measures. Interaction (T × C) [F_T×C_ = 7.85; *gl* = 910; η^2^ = 0.009; *p* = 0.005; 1-β = 799]. The simple effects T × C [T] (simple effects of the academic Course in each experimental group) were in EG [F_C_ = 10.58; *gl* = 910; η^2^ = 0.011; *p* = 0.001; 1-β = 0.901; MD ^*^4°P-3°P = 0.789], in the CG there were no statistically significant effects. The simple effects T × C [C] (simple effects of the Treatment in each academic year) were in 3°P [F_T_ = 126.30; *gl* = 910; η^2^ = 0.122; *p* = 0.000; 1-β = 1; MD ^*^EG-CG = 2.532], in 4°P [F_T_ = 45.30; *gl* = 910; η^2^ = 0.047; *p* = 0.000; 1-β = 1; MD ^*^EG-CG = 1.601].

#### Conclusions derived from the analysis with the complete data

The ANOVA results of the post-PR variable have highlighted that the Treatment has been effective with a moderate effect size and that Sex and Course variables explain part of the variance observed in the post-PR measure, however, its effect size is small, due to the fact that the sex variable is smaller. The ANOVA_ChS_ shows that the significant change has only been experienced by the EG. The 3°P students have been the most benefited by the treatment. It has been shown that the change observed in the CG is a product of maturation and has not been statistically significant.

#### Generation of missing data conditions

We have planned to lose data in five different conditions under two mechanisms of loss of data (hereafter McL), MCAR and MAR. In each of them we have planned to lose data in four percentage rates of data loss (hereafter PdL) 10, 20, 30, and 40% Addenda 3^*^.

We have manipulated the following conditions:

MCAR: completely random loss has been caused based on the total sample size without taking into account the EG and CC groups, Sex or Academic Course.

MAR1a: we have used the variable Sex to cause the loss of data. For each of the LpR, we plan to lose 80% of boys and 20% of girls.

MAR1b: we have used the pre PR measure to cause data loss. We calculate the P_25_ and P_75_ percentiles of the variable pre PR and segmented the variable into three categories, below P_25_, above P_75_ and between both percentiles. Subsequently, for each of LpR, the percentage of loss was 75% of those who had a measure below P_25_, 23% of those who had a measure in the middle segment, and 2% of those who had a measure above P_75_.

MAR2a and MNAR2b: the data loss has been the inverse of the manipulated in conditions MAR1a and MAR1b.

It is logical to think that those students who have worse initial performance will experience a poorer response to treatment. It is also logical to think that the response to treatment is will be similar in both boys and girls. Thus, the data loss MAR1b and MAR2b will have a more pernicious effect on the result than the data loss MAR1a and MAR2a Addenda 4^*^.

#### Data analysis

Having into account the McL in the 4 PdL, we examined the consequences of data loss on the empirical result of four statistics given by the two analysis models, ANOVA and ANOVA_ChS_ using the PR variable. In the ANOVA only on the main effect of the Treatment, and in the ANOVA_ChS_ only on the simple effect T × C [C] on the 3°P group. The observed statistics are the mean square error (MSe), F, η^2^, and MD.

In the first place we will observe what happens in each of the McL in terms of the PdL through the empirical value of the statistics and the percentage of bias (Addenda 5^*^) that occurs with respect to the empirical value obtained in the set of complete data (top left and top right of Tables 2, 3 in Supplementary Material respectively). Second, we will highlight the global differences between the McL's through the calculation of the mean value (M), standard deviation (SD) and coefficient of variation (CV) calculated in the set of the PdL Addenda 6^*^.

#### Results obtained in simulated situations

##### With respect to the ANOVA of the variable post-PR

MSe: when the McL is MCAR the MSe remains close to the MSe obtained with the CD's, and stable (very small SD), for all PdL. However, it undergoes a progressive reduction with respect to the estimate with CD (hereafter, w.r.e CD) as the PdL increases when the McL is MAR1a and MAR1b, in the latter the reduction is grater. The percentage of bias highlights this behavior more clearly. In this particular case, as the McL is more severe and w.r.e CD, the smaller is the MSe, the more vulnerable is its estimate on the PdL.

η2 and the MD: when the McL is MCAR both statistics stay close to the value obtained with the CDs. They do not experience any tendency based on the rate of loss. The mean in the set of loss rates is the same in both, η2 and the MD, with the values obtained with CD. When the McL is MAR1a both statistics experience a reduction in their value, and although they do not draw a trend based on the loss rate (at least in a clear way), they do experience greater variability than in MCAR. When the mechanism is MAR1b the result is even more sensitive to the loss rate (higher CV than in MAR1a), but very similar in both. However, although both, η^2^ and MD, have a lower average estimate w.r.e CD, MD experiences a greater reduction for PdL ≥30% (see percentage of bias).

F: in all McL the F value undergoes a progressive reduction of its value as the PdL increases. The reduction with respect to the CDs is greater when the McL is MAR1a and MAR1b in the rates 10, 20, and 30%. When the PdL is 40% the percentage of bias is the same in all McLs. The CV is high in the three McL.

If we now focus our attention on the McL MAR2a and MAR2b, the empirical results are inversed to those observed in the McL MAR1a and MAR1b. The effect exerted by the PdL in each McL is the same, but empirically, the MSe increases as the PdL increases, experiencing greater magnitude in MAR2b. In the same way, both η^2^ and DM are greater.

##### With respect to the ANOVA_*ChS*_

The results with respect to MSe and F are similar to those obtained in the ANOVA previously described. However, the results in η^2^ and MD present some nuances with respect to the results of ANOVA. The detailed results are shown in the Addenda 7^*^.

##### Substantive or clinical conclusions

The substantive conclusions of the effect of the Treatment when the McL is MAR1b and MAR2b vary in many nuances with respect to the conclusions derived from the analysis with the CDs, but in no case would they lead to conclusions opposite to those obtained with the CDs. The last section abounds in this aspect.

### Second research

#### Description and objective

Experimental research completely randomized to study the efficacy of two psychological therapies in the treatment of substance use disorder (SUD), acceptance and commitment therapy (ACT) and cognitive-behavioral therapy (CBT) (Villagrá et al., 2014). Out of 98 women in prison diagnosed with current substance use disorder, 50 agreed to participate in the study. The women's age ranged from 21 to 49 (*M* = 33.2, SD = 7.3). Randomization took place at prison using a random numbers table prior to the participants' transfer to the treatment programs (CBT, *n* = 19; ACT, *n* = 18; CG, *n* = 13). Assessment was carried out by two psychologists (one specialized in CBT and the other in ACT) who were in charge of interventions and measures. They had specific training in the methodology and the instruments used in this study. Ninety minute Tests were administered individually in the medical office to each inmate. Both interventions were conducted simultaneously, following a treatment protocol, and comprised 16 weekly group sessions. After treatment, all participants were evaluated by their therapist. Six months later a follow-up assessment was carried out. At the 6-month follow-up, 4 women from the CBT group, 3 women from the ACT group, and 2 women from CG had dropped out of study (18% of the sample, 8 for absolutely random causes, 5 were moved and 3 were released, only one person left due to non-random causes).

#### Data analysis

The first step to test the working hypothesis was to examine the possible existence of selection bias. Next, assuming that the causal model is a model of change (Judd and Kenny, 1981), we analyzed the pre-/post-test differences in scores to examine the effect of the treatments (ANOVA_ChS_). To determine whether the change undergone was maintained or disappeared over time, we analyzed the rate of change between follow-up and the initial measures.

The results of the research show that the women who received treatment benefited by the interventions. At post-treatment, CBT was more effective than ACT in reducing anxiety sensitivity, however, at follow-up, ACT was more effective than CBT in reducing drug use (43.8 vs. 26.7%) and improving mental health (26.4 vs. 19.4%).

In the quoted work you can find all details about the investigation and the results. In this case, to demonstrate how statistical results and substantive conclusions are altered when data loss occurs, we will only do so analysing three measures, ASI *Total*, ASI *Cognitiv*e and AAQ-II. The three variables describe by themselves the effect that the intervention exerted Addenda 8^*^.

With respect to the variables ASI *Total* and ASI *Cognitive*, the results are satisfactory if the subjects experienced a lower score after finishing the therapy, and with respect to the variable AAQ-II, if they experienced a higher score.

**Results obtained with the complete data in the selected variables** (see Adennda Tables 5, 6 in Supplementary Material).

Results ASI *Total*. After treatment only CBT had experienced a statistically significant change [F_PC_ = 5.52; *gl* = 47; η^2^ = 0.190; *p* = 0.007; 1-β = 902] distancing itself from GC, which had worse performance, and from ACT that did not undergo any change. At 6-months follow-up, the ANOVA_ChS_ shows that the change experienced at post-treatment is not maintained, and therefore there are no statistically significant differences. However, it is convenient to observe the estimated change scores, and note that GC does not change with respect at post-treatment, that CBT experiences a tendency to return to the initial values, and that ACT starts a trend toward improvement.

Results ASI *Cognitive*. After treatment, the two groups that received therapy experienced a statistically significant change [F_PC_ = 6.84; *gl* = 47; η^2^ = 0.225; *p* = 0.002; 1-β = 904] to a greater extent CBT, distancing themselves from CG, whose condition got worse. At 6-months follow-up, ANOVA_ChS_ shows that ACT continues to improve significantly [F_PC_ = 2.70; *gl* = 39; η^2^ = 0.122; *p* = 0.080; 1-β = 504]. CG does not get worse with respect to post-treatment. Again, CBT experiences a tendency to return to baseline after the 6-month treatment completion.

Results AAQ-II. See Addenda 9^*^.

#### Conclusions derived from the analysis with the complete data

The results obtained with the complete data in these three variables give us the same results as in the original research, and therefore can be conclude that ACT may be an alternative to CBT for treatment of drug abuse and associated mental disorders. In fact, at long-term, ACT may be more appropriate than CBT for women in prison with severe problems.

#### Generation of missing data conditions

Being the sample so small, it is illogical to plan a loss rate of 40%. If this were the case, the researcher would be probably doing things wrong. (see Addenda 10^*^). Because the previous example showed clear differences between the PdL 10 and 30%, we decided to manipulate only these two PdL. So, we planned to lose data under three different conditions under the McL MCAR and MAR.

MCAR: completely random loss has been caused in the total set of the sample without taking into account the ACT, CBT, and CG groups.

As discussed above, 49.9% of people who used drugs refused to participate in the research. It was found that most of them had been using drugs for many years, so was the case of the person who abandoned the research. For this reason we planned to lose data based on the variable “years of dependence.” This variable is distributed in a normal way (P_25_ = 10, P_50_ = 16.5, and P_75_ = 20.25 are its percentile values).

We planned two PdL MAR, 10 and 30%. In both conditions, the loss in each group was made according to the percentage of sample that each group represented of the total sample. Details are shown in the lower part of Table 1 in Supplementary Material.

MAR 10%: the 10% loss occurs only among subjects who had been consuming more than 20 years (above P75).

MAR 30%: loss occurs in the full range of the variable “years of dependence,” but the greatest amount was lost in those who had a longer period of dependence.

#### Data analysis

We examined the consequences of the data loss according to McL in both PdL on the empirical result of four statistics provided by the ANOVA_ChS_ model, MSe, F, η^2^, and DM, in the same way as we did in the first research.

**Results obtained in the simulated situations** (see Adennda Tables 5, 6 in Supplementary Material). In this case we show the empirical results and the percentage of bias, but not the M, SD, and CV in the set of PdL in each condition of McL because it is only done for 2 PdL and it would lose sense.

The results will be shown in a different way as we did in the first example. First we will comment on the empirical results and the statistical conclusions, and then, in block, we will comment on the bias that occurs in the estimation of MSe, F, η^2^, and MD. The small sample size forced us to focus attention, more intensively than in the previous example, on the substantive consequences of the statistical reading and on the variation of the magnitude of the means.

#### Statistical results and substantive or clinical conclusions

Variable ASI *Total*: at post-treatment (see Table 4, left in Supplementary Material) we observed that when PdL is 10% the statistical conclusion is the same as that obtained with CDs, whether McL is MCAR or MAR. When PdL is 30%, in both McL, MCAR, and MAR, we conclude that there are no statistically significant differences, opposite to what was found with CDs.

However, it should be added that only in MCAR with PdL 10% the substantive conclusion is the same as with CDs (observe the change rates, hereafter ChR). Although in MAR with PdL 10% we arrive to the same statistical conclusion, we should admit, reading the ChR, that both ACT and CG get worse in the same way.

The conclusions we reached were the same for both studies: The one with complete data and the 6-month follow-up with manipulated conditions of data loss. There is not a statistically significant change in any of the groups. However, it is necessary to comment on the reading of CDs the tendency toward the improvement that occurs in ACT and the tendency to lose the effect gained with the treatment in CBT. Again we observe that it is only true for MCAR with PdL 10%. In the MCAR condition with PdL 30% we would conclude that GC remains almost as at the beginning, in the MAR condition with PdL 10% we would conclude that ACT gets worse, and in the MAR condition with PdL 30%, we would conclude that the three groups practically behave in the same way.

Variable ASI Cognitive: at post-treatment (see Table 5, left in Supplementary Material) we observe that when PdL is 10% the global statistical conclusion is the same as that obtained with CDs, whether McL is MCAR or MAR. However, although ChR are very similar to those obtained with CDs, and in a substantive way we could conclude in the same way in both McL, in MCAR the differences between ACT and CG are not shown. When PdL is 30%, in both McL, MCAR and MAR, we conclude that there are no statistically significant differences, opposite to what was found with CDs. However, it should be noted that only in MCAR the substantive conclusion is the same as with CDs (observe the CHR). When McL is MAR we conclude that CBT gets bad, even worse than GC.

At the 6-month follow-up of manipulated condition of data loss we reached the same conclusions as we did with CDs. However, when McL is MCAR we arrive to the same conclusions. When McL is MAR and PdL is 10%, although we conclude that there are differences between ACT and GC, as with CDs, we also conclude that there are differences between CBT and CG, and this is so because the GC continues to experience a progressive deterioration after 6 months (that is not appreciated in CD).

Variable AAQ-II. See Addenda 11^*^.

**Evaluation of the bias in the empirical results** of MSe, F, p, η^2^, and 1-β. (see Tables 5, 6, bottom in Supplementary Material).

Observing the percentage of bias that occurs in the ANOVA_ChS_ (post-pre) estimates for ASI *Total* and ASI *Cognitive* variables (Table 4, left in Supplementary Material), we note that they experience the same pattern of bias. The bias suffered by MSe is greater in McL MAR than in McL MCAR, and is higher in PdL 30% than in PdL 10%. The F_PC_, p, η^2^, and 1-β statistics are more vulnerable to PdL than to McL because they experience a higher percentage of bias (much higher) in PdL 30% than for PdL 10% in both McL, however, for the variable ASI *Cognitive* bias is always greater in McL MAR, and in Total *ASI* in MCL MCAR. In both ASI *Total* and *Cognitive*, the sign of the bias is the same.

Observing the percentage of bias that occurs in the ANOVA_ChS_ estimates (6m-pre) in the ASI *Total* and ASI *Cognitive* variables (Tables 4, 5, right in Supplementary Material), except in very few exceptions, we note that in each McL condition the percentage of bias is greater when PdL is 30%, than when PdL is 10%. It is also observed that when the McL is MAR they experience greater bias than when it is MCAR.

With regard to the AAQ-II variable see Table 6 in Supplementary Material, Addenda 12^*^.

### Molar conclusions and particular nuances about the empirical and substantive results derived from the loss conditions manipulated in both investigations

It should be pointed out that:

1.—We have verified that MSe experiences variability both in function of PdL and in function of McL. The greater the bias the greater the PdL and the bias it is also greater if the McL is more severe. In the condition that both PdL and McL are more aggressive, the bias that MSe suffers is even greater. This happens in both models of data analysis, ANOVA and ANOVA_ChS_, in the first research, and in the ANOVA_ChS_ in the second research.

2.—We have verified that for the ANOVA model the bias experienced by η^2^ and MD suffers the same tendency as the bias experienced by MSe, although to a lesser extent. The variability suffered by η^2^ and MD in ANOVA_ChS_ is milder.

3.—In both investigations we have verified that McL MCAR is the less aggressive, even innocuous when PdL is ≤ 30% in the first research (see some exception in Addenda 13^*^). However, in the second research, in MCL MCAR, a PdL 30% affects the result in a very important way. This inevitably highlights the importance of the sample size and arrangement. The first research has a very large and very homogeneous sample size and the second research has a very small and very heterogeneous sample size (see some reasons in Addenda 14^*^). This is the reason why, even when McL is MCAR, sometimes it is not inocuos for all variables with a loss of 10%, (see results ASI *Cognitive*, second research), and when the loss is 30% the effects are even more aggressive (see results ASI *Total* and ASI *Cognitive*, second research).

4.—In both investigations we have verified that the MAR mechanism is more aggressive than MCAR. We have also checked the following:

—Their aggressiveness is directly related to PdL, that is, the results are more affected when the loss rate gets higher. We have seen that the model that best fits the data is different from that obtained with CDs, some-times in both investigations (see Addenda 15^*^). Again, we have to consider the importance of sample size and composition. While in the research carried out in schools the effect of the variable treatment is not modified when the model changes, and neither the substantive nor clinical conclusions (we have obviously seen that there are different nuances), in the research done with drug-dependent women the statistical result is radically modified, and the substantive conclusions too.

—The pernicious effects of data loss are greater when McL is MAR2 than when McL is MAR1. The reason is very simple. In MAR1 the loss of data is conditioned to the Sex variable, and although there are differences between boys and girls in the response to the dependent variable (the response is better and more homogeneous in girls), the response to Treatment is the same in boys and girls (see Addenda 16^*^). However, in MAR2 in the first research, and in MAR in the second research, something very different happens. The loss of data is conditioned to the initial PR measurement in the first research, and in the second research to the number of years that the women in prison have been consuming, and both variables are capable of determining the decision to abandon the investigation and thereby provoke selection bias exerting a very negative effect on both statistical results and on substantive or clinical results, see Addenda 17^*^. In MAR1b the available subjects are the students with the best initial PR measurements, their response to the treatment is expected to be more homogeneous, and that is what happens, that is why the MSe is very small with respect to CDs. Because the most advantaged subjects remain in both groups, EG and CG, η^2^ and MD are lower than those found with the CDs. In MAR2b the available subjects are the students with worse initial PR measurements, their response to the treatment is more heterogeneous, and that is why the MSe is greater with respect to CDs. Because the subjects are less advantaged in both groups, EG and CG, η^2^, and MD are greater than those found with CDs. In MAR in the second research, this is appreciated with both PdL, but much better with PdL 30%. The available subjects have been using drugs for less time and have a more homogeneous response to treatment, which is reflected in a smaller MSe than that found with CDs, and clinical conclusions that are absolutely contradictory to those that emerge from the results found with the complete data. This is shown, in a very aggressive way, in the results and conclusions of ASI *Total* and ASI *Cognitive* variables, but it does not happen so drastically for the AAQ-II variable. A statistical reason that may explain this is that ASI variables positively correlate with “years of dependence” variable (although only with ASI *Total* in a statistically significant way), but the variable AAQ-II does not. It is true that there may be substantive reasons that could explain the behavior of this variable, but they are not the aim of the present research.

5.—Points 3 and 4 above show the serious effects that the loss of data can have on statistical results, and the distorted reality conclusions we arrive to when the loss of data is MNAR. When we have manipulated the conditions of data loss in the two previous examples, we have defined the MAR conditions, as MAR conditions, because we know to which variable the losses are conditioned. If these losses occur in our research and we do not know the causes that determine them, the losses MAR1a and MAR1b are MAR losses, and it is possible to extract the information they provide from the complete data set. But the losses MAR1b, MAR2b in the first research, and the MAR losses in the second one, are MNAR losses and the data analysis gets highly complicated. Success will depend on our skill when choosing the variables that determine the losses, and that we have taken a record of them that allows us to introduce them in the models of imputation or maximum authenticity (in this way we convert a MAR loss into an MNAR loss), in addition loss of data from other variables may depend on different causes, see Addenda 18^*^. The sample size is very important too (these techniques have a better behavior when the sample size is large) and it is also important the sensitivity of the dependent variables to other variables also related to the treatment (if the sample is homogeneous the analysis will be easier and the results will be better). In any case, we are obliged to perform complex sensitivity analysis. And even then, the result will always be uncertain.

### Final suggestion

Where there are no data, and should there be…there is uncertainty only…now I suggest that you should read again the experts' recommendations and advice. It is important that you take them into account next time you have to investigate.

It was notified.

## Conclusions

The universe of missing data is large and complex. We have attempted to provide a simple, coherent and reasoned presentation of this enormous problem in order for it to be useful for applied researchers. With our paper, we attended to one of the requests presented in the manual The Prevention and Treatment of Missing Data in Clinical Trials (National Research Council, 2010, Recommendation 17, pp. 113–114), which is to emphasize the importance of becoming familiar with both terminology and current methods used in this field, providing training, and keeping abreast of new technique developments.

Although we have avoided all mathematical formulation, we could not avoid the appearance of terms with which the applied researcher may not be too familiar (*maximum likelihood, Bayesian estimation, Monte Carlo simulation, sensitivity analysis*, etc,) Nevertheless, we hope to have piqued your curiosity, but above all, we hope to gain your commitment to take responsibility and to be on guard with the aim of preventing and limiting data loss. Only in this way, if despite everything you are not able to avoid the loss of data, you will have the opportunity to address the problem technically (analytically) in a more satisfying way to further advance the development of knowledge.

We have noted that there is no perfect or infallible way to deal with the problem once the research has already been carried out, but without a doubt the best way to approach the objectives of our study, the best way to test the hypotheses will be by combining three aspects: the humility to recognize the problem, the time to devote to studying our database in depth, and the decisiveness to seek the methodological expertise of an expert in order to attempt to solve the problem together.

## Author contributions

MF-G and GV-S developed the initial idea and design of the work and wrote the article. PL-R and ET-H were in change of drafting the manuscript and revised the manuscript critically for important intellectual content. All four authors provided final approval of the version to be published and agreed to be accountable for all aspects of the work in ensuring that questions related to the integrity of any part of the work were appropriately resolved. The four authors have read and followed the Frontiers in Psychology Instructions for Authors, and the paper has been seen and approved by all of us.

### Conflict of interest statement

The authors declare that the research was conducted in the absence of any commercial or financial relationships that could be construed as a potential conflict of interest.
