# Supplementary material for: The (Ir)Responsibility of (Under)Estimating Missing Data
Source: Front Psychol. 2018 Apr 20;9:556. doi: 10.3389/fpsyg.2018.00556 (PMC5920143; doi:10.3389/fpsyg.2018.00556)
Supplement: Supplementary file 1 [file Presentation1.pdf]

Addenda

1* Cuetos, F., Rodríguez, B., Ruano, E., & Arribas, D. (2007). *PROLEC-R, Batería de Evaluación de los Procesos Lectores, Revisada*. Madrid: TEA.

2* It is foreseeable that the variables Sex and Academic year exert an additive or moderating effect (interaction) on the treatment effect, for this reason the "gross" effect of the treatment on the post-PR measure was tested by the Variance Analysis Model, ANOVA (2x2x2) [EG, CG; boys, girls; 3ºP, 4ºP]. Because it was 3-month treatment, it is expected that the CG undergoes a slight change between the pre and post measure due to the effect of maturation, however, it is expected that this change will not be statistically significant. To verify this hypothesis, we used an ANOVA analysis on the change scores (post PR-pre PR), hereafter $ANOVA_{ChS}$ (2x2x2).

3* Peng, Harwell, Liou and Ehman (2006) and Peugh and Enders (2004) reviewed missing data reported in a large number of Education and Psychology Research Journals. Peng et al., (2006) found that the lost data rate ranged between 1% and approximately 67%, and Peugh and Enders (2004) found that it was between 26% and 72%. We think, therefore, that the 4 missing data rates that we manipulate represent a reasonable range on the percentage of data that is possible to lose in real research. Added to this, it must be said that these missing data rates are usually studied by simulation processes in methodological research when validating the effectiveness of this kind of analysis procedures.

Peng, C. Y. J., Harwell, M., Liou, S. M., & Ehman, L. H. (2006) Advances in missing data methods and implications for educational research. In S. S. Sawilowsky (Eds.) *Real Data Analysis* (pp. 31-78). Charlotte, North Carolina: Information Age Pub.

Peugh, J. L., & Enders, C. K. (2004) Missing data in educational research: A review of reporting practices and suggestions for improvement. *Review of Educational Research, 74*(4), 525-556.

4* Many research papers have shown that Primary School boys are less disciplined than girls and have a worse performance in variables related to learning. On the other hand, it is logical to think that students who have a poor initial measure also have a poor response to treatment. That said, because the research has already been done, we know that the response to treatment occurs with the same intensity in boys and girls, even though boys have a worse performance than girls in the dependent variable pre PR. We also know that the students with better performance in the pre PR measure experience a more positive and homogeneous response to treatment than the students who have worse performance in the pre PR measure. Thus, the loss of data MAR1b and MAR2b will have a more aggressive effect on the results than the loss of data MAR1a and MAR2a. This is the reason why we have manipulated losing data in this way and that will help us to understand the statistical and substantive or clinical results that the loss of data cause in this specific study.

5* Percentage of bias was calculated as the ratio of the difference between the incomplete data estimate and the complete data estimate divided by the complete data estimate. That is why, if the result is negative, it is because the value of the empirical statistic in the manipulated conditions is lower (otherwise, it would be larger). The sign of the bias is important, but it is more important the bias magnitude that takes place.

6* The average value allows us to compare the deviation that occurs with respect to the empirical value obtained when using complete data. The standard deviation allows us to examine the sensitivity that occurs on one McL in function of the loss rate. The coefficient of variation allows us to examine in which McL, in which observed statistic and in which analysis model (ANOVA or $ANOVA_{ChS}$) the vulnerability or sensitivity to the PdL is higher. Due to the fact research work has been very carefully done and because we know the results obtained with the complete data, it is possible to have a logical and coherent explanation of all the results found. And they fit well both with the methodological literature and with the substantive literature as it will be later shown in the text. In order to better understand this point, we will first focus on the results found in the MCAR, MAR1a and MAR1b conditions, and then in the MAR2a and MAR2b results.

7*<u>With respect to the ANOVA on the change scores. See Table 3</u>

MSe: when the McL is MCAR and MAR1a, the empirical estimation of the MSe remains close to the MSe obtained with the CDs, and we could say that it is relatively stable for all PdL. However, when the McL is MAR1b, the MSe undergoes a progressive reduction w.r.e CD as it increases the PdL. The percentage of bias highlights this behavior more clearly. In this particular case, the estimate of the MSe moves away from the MSe w.r.e CD and depends on the PdL (> CV) only when the McL is MAR1b.

$\eta 2$ and the MD: when the McL is MCAR both statistics stay close to the value obtained with the CD. They do not experience any tendency based on the PdL. The mean in the set of loss rates practically coincides in both, $\eta 2$ and the MD, with the values obtained with the CD. When the McL is MAR1a both statistics experience an increase in their average value, and although they do not draw a clear trend based on the rate of loss, they do experience greater variability in function of it than in MCAR. When the mechanism is MAR1b the result is even more sensitive to the loss rate (higher CV than in MAR1a). This happens to a greater extent in $\eta 2$ than in MD (there is a big difference in CV). However, although both have a lower average estimate w.r.e CD, both move away from that value to the same extent as under the McL MAR1a (compare percentages of bias).

F: in all McL the F value undergoes a progressive reduction of its value as the PdL increases. The reduction with respect to the CD is greater when the loss mechanism is MAR1a and MAR1b in the rates 10%, 20% and 30%. When the loss rate is 40% the bias rate is the same in all loss mechanisms. The CV is high in the three McL.

If we focus on the McL MAR2a and MAR2b, we observe that the average value of the MSe increases with respect to what happens in the analysis of the CD as it increases the PdL, experiencing greater increase in MAR2b. However, the measured values of $\eta 2$ and MD experience very little variation.

8* ASI, *Anxiety Sensitivity Index* (Peterson & Reiss, 1992) and AAQ- II, *Acceptance and Action Questionnaire II* (Hayes, Follette, and Linehan, 2004).

Hayes, S. C., Follette, V. M., & Linehan, M. M. (2004). *Mindfulness and acceptance: Expanding the cognitive-behavioral tradition*. New York: The Guilford Press.

Peterson, R. A., & Reiss, R. J. (1992). *Anxiety Sensitivity Index Manual* (2nd ed.). Worthington, OH: International Diagnostic Systems.

9* Results AAQ-II. The $ANOVA_{ChS}$ showed that the two groups that received therapy experienced a statistically significant change at posttreatment [F_{PC}=7.30; *gl*= 47; η^2=.237; *p*=.002; 1-β=922]. This change was greater for ACT than for CBT, both distancing themselves from CG, which condition got worse. At the 6-months follow-up, GC maintained the same impoverished state that it had reached at posttreatment, ACT continued to improve significantly and CBT lost much of the achievement reached after therapy [F_{PC}=11.20; *gl*=39; η^2=.365; *p*=.000; 1-β=995].

10* It was possible to test the treatment effect on the 50 subjects who participated in the research, but six months later 9 subjects dropped out. Only one of them was a non-random loss. The effort and care taken in measurements registration prevented from having a greater non-random loss. That was a difficult task.

11* Variable AAQ-II: Once the treatment is finished (Table 6, left) we observe that in all the conditions of loss, and for both PdL, the statistical conclusion is the same as for the CDs. However, the substantive reading is only the same when the PdL is 10% in both McL, for the rest of conditions the conclusions would have quite different nuances.
At follow-up we observed (Table 7, Right) that only when McL is MAR and PdL is 10% the global statistical conclusion is the same. Under the other conditions, we appreciate differences on both treatment groups with respect to CG. But the differences between ACT and CBT would not be appreciated and they are very important to prove the hypothesis of researchers.
However, this variable is less affected by the loss of data than the ASI *Total* and ASI *Cognitive* variables.

12* Regarding the AAQ-II variable, the $ANOVA_{ChS}$ (post-pre) estimators are more vulnerable to the PdL than to the McL because they experience a higher percentage of bias in 30% PdL than in 10% PdL for both McL, but the bias is not always greater in McL MAR than in McL MCAR. For the $ANOVA_{ChS}$ estimators (6m-pre) it is difficult to find a behavior pattern in the rate of bias both in terms of McL and in terms of PdL.

13* See in Table 3, the $ANOVA_{ChS}$ in the first study concludes that the best adjusted model is the additive model for a 40% PdL.

14* The first research works with a very large and homogeneous sample: students of the same age without serious learning difficulties who study and live in the same city. All of them receive treatment at school with the informed consent of parents and teachers. The second one works with a very small and heterogeneous sample. Women who participated in the research did so voluntarily. We should have in mind that the prison available population was 98 women, and half of them refused to participate. The variability of this sample can be seen in many variables, among them, the years of drug use, the type of drugs they consume, the living conditions they have had, the comorbidity with other physical and mental pathologies, etc.).

15* In the first research, the results of the ANOVA conclude that the model best adjusted when the PdL is 40% is not the same as the model explained by the CDs in MAR1a, MAR2a and MAR2b. The results of the $ANOVA_{PC}$ in MAR2a and MAR2b when the PdL is 30% arrive to the same conclusions. The same happens in the second research in the $ANOVA_{PC}$ with the results of the ASI *total* and ASI *Cognitive* variables.

16* Many studies have shown that both boys and girls have the same ability to learn. But, however, boys are less disciplined than girls at the age studied. For this reason, it is expected that in MAR1a, where we had mostly girls in the sample, the response to treatment would be more homogeneous than in MAR2a, where the available sample was composed by boys mostly, and this is what is reflected in MSe in both the ANOVA and the $ANOVA_{ChS}$, which in the first case, in MAR1a, the MSe is slightly lower than that found with the CDs and in the second case, in MAR1b, the MSe is slightly higher than that found with the CDs. This also explains why η2 and MD experience a very small bias with respect to the CDs in the ANOVA.

17* A low pre-PR measure may lead the students to consider that it is not worthwhile to undergo the treatment because they will not benefit from it. And therefore, the students may be discouraged and leave the research. A person who has been a drug addict for many years may think that at this point of life there is no use changing because there is nothing to win, and for this reason motivation is very low, and the chances of quitting are very high. Or simply, a person who has been using drugs for many years may not comply with the commitment "not to use drugs while the research goes on", and to avoid been caught does not take the blood test and thus we lose data (and therefore we could have an intermittent loss of data). All this causes selection bias. Selection bias has a very negative effect on both statistical and substantive or clinical results. This is what happens in MAR1b and in MAR2b in the first study, and in MAR in the second one.

18* We have seen that the causes of data loss do not have to be the same for all the variables that have lost data. This means that in the estimation of lost data for each variable we probably need to use different imputation models when choosing that way to approach the problem.

Demonstration. Tables

Table 1. Probability of missing data for post Prolec and pre Prolec in the first research, and for ASI Total, ASI Cognitive and AAQ-II in the second research at four missing rates in each condition of data loss.

Quasi-experimental research N=915

MmD	Way to make the losses	Loss rate			
		**10%**	**20%**	**30%**	**40%**
**MCAR**	**Random[2]**	**10[1] % [n_p=92]**	**17.8%[n_p=164]**	**28.9%[n_p=265]**	**39%[n_p=357]**
**MAR1a***		**10.2% [n_p=93]**	**20.7%[n_p=183]**	**31.2%[n_p=275]**	**40.1%[n_p=366]**
	**80% boys**	74	146	220	293
	**20% girls**	19	37	55	73
**MAR1b***		**10.3% [n_p=94]**	**20.1%[n_p=184]**	**30.3%[n_p=277]**	**40.2%[n_p=368]**
	**75% <P25**	69	138	207	275
	**P75<23%>P25**	23	42	64	85
	**2%>P75**	2	4	6	8

Experimental research N=50 (Initial and post treatment sample size)

MmD	Way to make the losses	Loss rate		
**MCAR**	**Random[3]**	**10% [n_p=5]**	**30% [n_p=15]**	
	**ACT [n=18; 36%]**	2	5	
	**CBT [n=19; 38%]**	2	6	
	**C [n=13; 26%]**	1	4	
**MAR**	**10% [n_p=5]**	ACT **[n_p=2; 36%]**	CBT **[n_p=2; 38%]**	GC **[n_p=1; 26%]**
	**100%>P75**	2	2	1
**MAR**	**30% [n_p=15]**	ACT **[n_p=5; 36%]**	CBT **[n_p=6; 38%]**	GC **[n_p=4; 26%]**
	**10% ≤P25**	1	1	1
	**30% Between P25 & P75**	1	2	1
	**60%≥P75**	3	3	2

Note. MmD= Mecanism missing data; N= Total sample size; n= sample size each group; n_p= number of students that are lost; 10%, 20%, 30% and 40%= missing rates planned; [1]= actual overall missing rates; P_{25} y P_{75}= percentile values of the pre variable pre Prolec; *= the losses MAR2a and MNAR2b occur inversely to losses MAR1 y MNAR1; [2]= random loss based on N without taking into account the groups GE y CC; [3]= loss in each group according to the percentage that represents N.

Table 2. AVAR of dependent variable post Prolec. Empirical estimation, percentage of bias in estimates and descriptive statistics in the set of missing rates.

	Empirical estimation of AVAR					Percentage of bias in estimates[2]			
	**Mr**	**CM_E**	**F_T**	**η^2**	**DM**	**CM_E**	**F_T**	**η^2**	**DM**
**CD**		**6.12**	**145.98**	**.138**	**1.99**				
**MCAR**	**10**	6.13	132.04	.139	2	.16	-9.55	.72	.50
	**20**	5.96	117.55	.136	1.95	-2.61	-19.48	-1.45	-2.01
	**30**	6.24	106.06	.141	2.04	1.96	-27.35	2.17	2.51
	**40**	5.98	86.80	.135	1.96	-2.29	-40.54	-2.17	-1.51
**MAR1a**	**10**	6.10	124.71	.132	1.93	-.33	-14.57	-4.35	-2.66
	**20**	6.35	107.68	.129	1.95	3.76	-26.24	-6.52	-1.96
	**30**	5.85	89.22	.123	1.82	-4.41	-38.88	-10.87	-8.34
	**40[1]**	5.72	84.80	.135	1.89	-6.54	-41.91	-2.17	-4.87
**MAR1b**	**10**	5.73	119.73	.128	1.842	-6.37	-17.98	-7.25	-7.44
	**20**	5.47	114.61	.136	1.871	-10.62	-21.49	-1.45	-5.98
	**30**	4.94	89.98	.123	1.683	-19.28	-38.36	-10.87	-15.43
	**40**	3.75	91.56	.144	1.603	-38.73	-37.28	4.35	-19.45
**MAR2a**	**10**	6.01	128.60	.136	1.95	-1.80	-11.91	-1.45	-2.01
	**20**	6.18	112.49	.134	1.96	.98	-22.94	-2.90	-1.51
	**30**	6.29	114.44	.153	2.14	2.78	-21.61	10.87	7.54
	**40[1]**	6.28	98.76	.153	2.15	2.61	-32.35	10.87	8.04
**MAR2b**	**10**	6.34	131.20	.138	2.07	3.59	-10.12	.00	4.02
	**20**	6.47	123.80	.145	2.10	5.72	-15.19	5.07	5.53
	**30**	6.68	113.84	.152	2.197	9.15	-22.02	10.14	10.40
	**40[1]**	7.10	120.69	.182	2.51	16.01	-17.32	31.88	26.13

Descriptive statistics in the set of missing rates

	CM_E=6.12			F_T=145.98			η^2=.138			DM=1.99		
	**M**	**SD**	**CV**	**M**	**SD**	**CV**	**M**	**DT**	**SD**	**M**	**SD**	**CV**
**CD**	**6.12**			**145.98**			**.138**			**1.99**		
**MCAR**	6.07	.13	2.14	110.61	19.10	17.27	.138	.003	2.17	1.99	.04	2.01
**MAR1a**	6.00	.27	4.50	101.60	18.31	18.02	.130	.005	3.85	1.90	.06	3.16
**MAR1b**	4.97	.87	17.51	103.97	15.39	14.80	.133	.009	6.77	1.75	.13	7.43
**MAR2a**	6.19	.13	2.10	113.57	12.20	10.74	.144	.010	6.94	2.05	.11	5.37
**MAR2b**	6.64	.33	4.97	122.38	7.20	5.88	.154	.019	12.34	2.22	.20	9.01

Note. Mr= Missing rates planned; CD= Complete data set (N=915); N=915; CM_E; F; η^2 y DM= Statistics defined in the text of the article; [1]= The model that best fits the data is not the Additive Model T+S+C (Treatment, Sex and Course), but the Additive Model T+C; [2]= Percentage of bias was calculated as the ratio of the difference between the incomplete data estimate and the complete data estimate divided by the complete data estimate; M = mean; SD = standard deviation; CV= coefficient of variation.

Table 3. $AVAR_{ChS}$ of dependent variable Prolec. Empirical estimation, percentage of bias in estimates and descriptive statistics in the set of missing rates.

	%P	CM_E	F_T	η^2	DM	CM_E	F_T	η^2	DM
	**Empirical estimation of $AVAR_{PC}$, F3P**					**Percentage of bias in estimates**			
**CD**		6.06	126.30	.122	2.532				
**W**		5.81	130.45	.137	2.628				
**MCAR**	**10**	5.98	111.46	.120	2.481	-1.39	-11.75	-1.64	-2.01
	**20**	6.17	111.72	.130	2.638	1.75	-11.54	6.56	4.19
	**30**	6.18	96.66	.124	2.569	1.91	-23.47	1.64	1.46
	**40[1]**	5.98	73.94	.121	2.549	-1.39	-41.46	-.82	.67
**MAR1a**	**10**	6.13	115.78	.124	2.580	1.09	-8.33	1.64	1.90
	**20**	6.26	93.88	.114	2.517	3.23	-25.67	-6.56	-.59
	**30**	6.00	104.47	.141	2.747	-1.06	-17.28	15.57	8.49
	**40**	6.07	81.55	.130	2.628	.10	-35.43	6.56	3.79
**MAR1b**	**10**	5.81	120.75	.129	2.594	-4.19	-4.39	5.74	2.45
	**20**	5.69	78.89	.098	2.260	-6.17	-37.54	-19.67	-10.74
	**30**	4.93	74.45	.105	2.386	-18.70	-41.05	-13.93	-5.77
	**40**	4.37	72.26	.117	2.466	-27.94	-42.79	-4.10	-2.61
**MAR2a**	**10**	6.13	111.83	.120	2.562	1.09	-11.46	-1.64	1.18
	**20**	6.27	103.06	.124	2.608	3.40	-18.40	1.64	3.00
	**30[1]**	6.38	74.45	.145	2.388	5.21	-41.05	18.85	-5.69
	**40**	6.07	90.47	.143	2.773	.10	-28.37	17.21	9.52
**MAR2b**	**10**	6.13	111.65	.120	2.507	1.09	-11.60	-1.64	-.99
	**20**	6.25	103.50	.125	2.547	3.07	-18.05	2.46	.59
	**30[1]**	6.50	87.40	.121	2.529	7.19	-30.80	-.82	-.12
	**40**	6.83	78.08	.126	2.586	12.63	-38.18	3.28	2.13

Descriptive statistics in the set of missing rates

	**CM_E=6.12**			**F_T=145.98**			**η^2=.138**			**DM=1.99**		
	**M**	**SD**	**CV**	**M**	**SD**	**CV**	**M**	**SD**	**CV**	**M**	**SD**	**CV**
**CD**	6.06			126.30			.122			2.532		
**W**	5.81			130.45			.137			2.628		
**MCAR**	6.08	.11	1.81	98.45	17.79	18.07	.12	.004	3.64	2.56	.06	2.52
**MAR1a**	6.12	.11	1.80	98.92	14.63	14.79	.13	.011	8.28	2.62	.10	3.72
**MAR1b**	5.20	.68	13.08	86.59	22.94	26.49	.11	.013	12.16	2.43	.14	5.78
**MAR2a**	6.21	.14	2.25	94.95	16.24	17.10	.13	.012	9.65	2.58	.16	6.13
**MAR2b**	6.43	.31	4.82	95.16	15.20	15.97	.12	.002	2.39	2.54	.03	1.32

Note. Ver Tabla 2. [1]= The model that best fits the data is the Additive Model T+C (Treatment and Course) instead of the non-additive model [T+C+(TxC)].

Table 4. AVAR$_{ChS}$. Empirical estimation and percentage of bias in estimates. *Total Anxiety Sensitivity Index.*

Empirical estimation. AVAR$_{ChS}$

McP		AVAR$_{ChS}$ (post-pre)						AVAR$_{ChS}$ (6m-pre)					
		**TC1**	**CM$_E$**	**F$_{PC}$**	**p**	**η^2**	**1-β**	**TC2**	**CM$_E$**	**F$_{PC}$**	**p**	**η^2**	**1-β**
**DC**	**ACT**	0	248.60	5.52	.007	.190	.902	-2.25	354.27	.758	.475	.037	.268
	**CBT**	-13.16						-6.73					
	**CG**	4.15						3.45					
	**Tukey**	ACT-CBT; CBT.CG						--------					
**MCAR10**	**ACT**	0	265.85	4.33	.019	.171	.823	-1.73	392.24	.602	.553	.033	.233
	**CBT**	-12.59						-5.54					
	**CG**	4.17						3.60					
	**Tukey**	ACT-CBT; CBT-CG						--------					
**MCAR30**	**ACT**	-3.77	251.82	2.085	.141	.115	.531	-5.92	433.77	.482	.623	.034	.204
	**CBT**	-12						-8.30					
	**CG**	1.67						1.12					
	**Tukey**	--------						--------					
**MAR10**	**ACT**	3.12	197.88	6.37	.004	.233	.935	1	288.98	1.405	.259	.074	.405
	**CBT**	-11.35						-4.93					
	**CG**	5.17						6.80					
	**Tukey**	ACT-CBT; CBT-CG						--------					
**MAR30**	**ACT**	4.31	167.23	2.70	.082	.144	.630	-1.27	263.32	.114	.892	.009	.124
	**CBT**	-6.62						1.91					
	**CG**	3.22						1.71					
	**Tukey**	--------						--------					

Percentage of bias in estimates. AVAR$_{ChS}$

	AVAR$_{ChS}$ (post-pre)					AVAR$_{ChS}$ (6m-pre)				
	**CM$_E$**	**F$_{PC}$**	**p**	**η^2**	**1-β**	**CM$_E$**	**F$_{PC}$**	**p**	**η^2**	**1-β**
**DC**	248.60	5.52	.007	.190	.902	354.27	.758	.475	.037	.268
**MCAR10**	6.939	-21.56	171.43	-10.00	-8.76	10.718	-20.58	16.42	-10.81	-13.06
**MCAR30**	1.295	**-62.23**	**1914.29**	**-39.47**	**-41.13**	**22.441**	**-36.41**	**31.16**	-8.11	**-23.88**
**MAR10**	**-20.402**	15.40	-42.86	22.63	3.66	-18.429	85.36	-45.47	**100.00**	51.12
**MAR30**	**-32.731**	**-51.09**	**1071.43**	**-24.21**	**-30.16**	**-25.673**	**-84.96**	**87.79**	-75.68	**-53.73**

Note. ACT = acceptance and commitment therapy; CBT = cognitive-behavioral therapy; CG = control group.TC1 and TC2 = change rate between the post and pre measurements, and between the measurement recorded 6 months after the treatment and the pre measurement, respectively.

Table 5. AVAR$_{ChS}$. Empirical estimation and Percentage of bias in estimates. *Cognitive* subscale of *Total Anxiety Sensitivity Index.*

Empirical estimation. AVAR$_{ChS}$

		AVAR$_{ChS}$ (post-pre)						AVAR$_{ChS}$ (6m-pre)					
		**TC1**	**CM$_E$**	**F$_{PC}$**	**p**	**η^2**	**1-β**	**TC2**	**CM$_E$**	**F$_{PC}$**	**p**	**η^2**	**1-β**
**DC**	**ACT**	-2.61	38.93	6.84	.002	.225	.904	-3.38	53.79	2.70	.080	.122	.504
	**CBT**	-5.26						-2.67					
	**CG**	3						2.91					
	**Tukey**	CBT-CG; ACT-CG						ACT-GG					
**MCAR10**	**ACT**	-2.25	39.46	5.42	.0087	.205	.819	-3.13	58.99	2.18	.128	.111	.551
	**CBT**	-4.94						-2.62					
	**CG**	2.83						3					
	**Tukey**	CBT-CG						--------					
**MCAR30**	**ACT**	-2.92	39.33	2.94	.067	.155	.534	-4	58.97	1.73	.196	.114	.463
	**CBT**	-4.31						-3					
	**CG**	2.11						2					
	**Tukey**	--------						--------					
**MAR10**	**ACT**	-2	35.87	6.69	.003	.242	.895	-2.64	46.37	3.57	.039	.170	.746
	**CBT**	-4.53						-2.14					
	**CG**	3.67						4.30					
	**Tukey**	CBT-GT; ACT-GT						ACT-CG; CBT-CG					
**MAR30**	**ACT**	-2.08	27.50	2.18	.129	.120	.414	-2.82	45.58	.967	.393	.069	.308
	**CBT**	2.38						-1.27					
	**CG**	2						1.71					
	**Tukey**	--------						--------					

Percentage of bias in estimates. AVAR$_{ChS}$

	AVAR$_{PC}$ (post-pre)					AVAR$_{PC}$ (6m-pre)				
	**CM$_E$**	**F$_{PC}$**	**p**	**η^2**	**1-β**	**CM$_E$**	**F$_{PC}$**	**p**	**η^2**	**1-β**
**DC**	38.93	6.84	.002	.225	.904	53.79	2.70	.080	.122	.504
**MCAR10**	1.361	-20.76	335.00	-8.89	-9.40	**9.667**	-19.26	60.00	**-9.02**	**9.13**
**MCAR30**	1.027	**-57.02**	**3250.00**	**-31.11**	**-40.93**	9.630	**-35.93**	**145.00**	-6.56	-8.33
**MAR10**	**-7.860**	-2.19	50.00	7.56	-1.00	-13.794	32.22	-51.25	39.34	**47.82**
**MAR30**	**-29.360**	**-68.13**	**6350.00**	**-46.67**	**-54.20**	**-15.263**	**-64.19**	**391.25**	**-43.44**	-39.09

Note. See Table 5.

Table 6. AVAR$_{ChS}$. Empirical estimation and percentage of bias in estimates. *Acceptance and Action Questionnaire II.*

Empirical estimation. AVAR$_{ChS}$

		**AVAR$_{ChS}$ (post-pre)**						**AVAR$_{ChS}$ (6m-pre)**					
		**TC1**	**CM$_E$**	**F$_{PC}$**	**p**	**η^2**	**1-β**	**TC2**	**CM$_E$**	**F$_{PC}$**	**p**	**η^2**	**1-β**
**DC**	**ACT**	14.28	247.60	7.30	.002	.237	.922	18.69	188.37	11.20	.000	.365	.995
	**CBT**	12						7.27					
	**CG**	-6.15						-6.73					
	**Tukey**	ACT-CG; CBT-CG						ACT-CG; CBT-CG; ACT-CBT					
		**DM**	**CM$_E$**	**F$_{PC}$**	**p**	**η^2**	**1-β**	**DM**	**CM$_E$**	**F$_{PC}$**	**p**	**η^2**	**1-β**
**MCAR10**	**ACT**	13.88	261.72	5.85	.006	.218	.849	18.20	206.41	9.23	.001	.345	.985
	**CBT**	10.47						8					
	**CG**	-6.25						-7					
	**Tukey**	ACT-CG; CBT-CG						ACT-CG; CBT-CG					
**MCAR30**	**ACT**	19.23	234.81	9.97	.000	.384	.975	21.25	184.85	11.31	.000	.456	.995
	**CBT**	6.85						12					
	**CG**	-10.44						-8.13					
	**Tukey**	ACT-CG; CBT-CG						ACT-CG; CBT-CG					
**MAR10**	**ACT**	13.69	247.27	6.74	.003	.243	.897	19.07	167.47	14.53	.000	.454	.999
	**CBT**	10.53						7.93					
	**CG**	-7.10						-9.80					
	**Tukey**	ACT-CG; CBT-CG						ACT-CG; CBT-CG; ACT-CBT					
**MAR30**	**ACT**	11.62	192.51	5.15	.011	.244	.788	16.91	181.81	6.41	.005	.331	.929
	**CBT**	8.85						8.64					
	**CG**	-6.78						-6.43					
	**Tukey**	ACT-CG; CBT-CG						ACT-CG; CBT-CG					

Percentage of bias in estimates. AVAR$_{ChS}$

	**AVAR$_{ChS}$ (post-pre)**					**AVAR$_{ChS}$ (6m-pre)**				
	**CM$_E$**	**F$_{PC}$**	**p**	**η^2**	**1-β**	**CM$_E$**	**F$_{PC}$**	**p**	**η^2**	**1-β**
**DC**	247.60	7.30	.002	.237	.922	188.37	11.20	.000	.365	.995
**MCAR10**	**5.703**	-19.86	200.00	-8.02	**-7.92**	**9.577**	**-17.59**	-88.89	-5.48	-1.01
**MCAR30**	-5.166	**36.58**	**-100.00**	**62.03**	5.75	-1.869	.98	**-100.00**	**24.93**	.00
**MAR10**	-.133	-7.67	50.00	2.53	-2.71	**-11.095**	29.73	**-100.00**	**24.38**	.40
**MAR30**	**-22.250**	**-29.45**	**450.00**	**2.95**	**-14.53**	-3.483	**-42.77**	-44.44	-9.32	**-6.63**

Note. See Table 5.
